# Sodium-Glucose Cotransporter 2 (SGLT2) Inhibitors in Heart Failure With Preserved Ejection Fraction: A Systematic Review

**DOI:** 10.7759/cureus.86368

**Published:** 2025-06-19

**Authors:** Amir Saeed, Bilal Younas, Ali Rohan, Usman Haider, Asad Mukhtar, Marriam Nazir, Maryam

**Affiliations:** 1 Acute Medicine, Norfolk and Norwich University Hospital, Norwich, GBR; 2 Cardiology, Mardan Medical Complex, Mardan, PAK; 3 Respiratory Medicine, Sunderland Royal Hospital, Sunderland, GBR; 4 Internal Medicine, University College of Medicine and Dentistry, Lahore, Lahore, PAK; 5 Medicine, Naas General Hospital, Health Service Executive, Naas, IRL; 6 Internal Medicine, Faisalabad Medical University, Faisalabad, PAK; 7 Medical Oncology, Allied Hospital Faisalabad, Faisalabad, PAK; 8 Internal Medicine, Sharif Medical and Dental College, Lahore, PAK

**Keywords:** cardiovascular death, exercise capacity, hfpef, hospitalization, kansas city cardiomyopathy questionnaire, renal outcomes, sglt2 inhibitors

## Abstract

Heart failure with preserved ejection fraction (HFpEF) is a complex syndrome characterized by impaired ventricular filling and increased heart failure hospitalizations. Sodium-glucose cotransporter 2 (SGLT2) inhibitors have demonstrated cardiovascular and renal benefits in various heart failure populations, but their effects on HFpEF remain an area of growing interest. This study aims to evaluate the impact of SGLT2 inhibitors on key clinical outcomes in patients with HFpEF, including cardiovascular death, hospitalization for heart failure, exercise capacity, symptoms (as measured by the Kansas City Cardiomyopathy Questionnaire (KCCQ)), kidney disease progression, and other renal outcomes. A systematic review of randomized controlled trials (RCTs) assessing the effects of SGLT2 inhibitors (empagliflozin, dapagliflozin, sotagliflozin, canagliflozin, and ertugliflozin) in HFpEF patients was conducted. This systematic review was conducted per the Preferred Reporting Items for Systematic Reviews and Meta-Analyses (PRISMA) principles. The literature was searched using open-access, full-text English papers from January 2015 to April 2025 across PubMed, Embase, and the Cochrane Library. A total of 108 articles were retrieved through the initial search. After screening and checking for eligibility according to the pre-specified inclusion criteria, the methodological quality was assessed in 17 included studies using the Mixed Methods Appraisal Tool (MMAT) score. The MMAT Score 4 indicates a medium risk of bias (ROB), and the MMAT Score 5 indicates a low ROB. Ten studies had low ROB and were classified as "high quality." Seven had uncertain ROB, lowering the evidence by one point to "moderate quality," while one study had a high ROB. SGLT2 inhibitors were associated with significant reductions in cardiovascular death and heart failure-related hospitalizations. Improvements in KCCQ total symptom scores were observed, indicating enhanced patient-reported outcomes. The renal benefits of SGLT2 inhibitors were evident, with a reduction in kidney disease progression and a marked decrease in cardiovascular-related renal outcomes.

## Introduction and background

Heart failure with preserved ejection fraction (HFpEF) represents a unique subtype of heart failure, distinguished by maintained systolic function and a complex, comorbidity-driven pathophysiology [1,2]. HFpEF accounts for approximately 50% of heart failure cases and presents a growing burden with no proven disease-modifying treatment [3,4]. HFpEF is a major dilemma in cardiology because, despite its high prevalence and symptom burden, it lacks effective, evidence-based treatments, unlike heart failure with reduced ejection fraction (HFrEF), which has several proven therapies. Its complex, multi-system pathophysiology further complicates diagnosis and management [5].

While both HFpEF and HFrEF involve symptoms like congestion and exercise intolerance, they differ significantly in heart function and present distinct diagnostic and therapeutic challenges. HFpEF maintains a preserved ejection fraction (≥50%), whereas HFrEF involves a reduced ejection fraction (<50%) [6-8]. Patients with HFpEF exhibit a preserved left ventricular ejection fraction (≥50%) yet suffer from symptoms such as dyspnea, fatigue, and exercise intolerance, largely driven by increased myocardial stiffness and systemic comorbidities [9,10]. The pathophysiology of HFpEF is multifaceted, involving systemic inflammation, endothelial dysfunction, and diastolic impairment, often exacerbated by coexisting conditions like hypertension, obesity, and type 2 diabetes mellitus (T2DM) [11].

In recent years, sodium-glucose cotransporter 2 (SGLT2) inhibitors have emerged as a promising therapeutic option. Initially developed for glycemic control, these agents have demonstrated cardiovascular benefits that extend beyond blood glucose regulation [12,13]. SGLT2 inhibitors act through several mechanisms potentially relevant to HFpEF. These include reducing cardiac preload via osmotic diuresis and natriuresis, improving myocardial energy metabolism through enhanced ketone body utilization, and modulating systemic inflammation and oxidative stress [14-16]. Additionally, they support renal function and reduce body weight and epicardial fat, factors that may further alleviate cardiac strain [17-19].

Emphasizing that HFpEF accounts for over 50% of heart failure cases yet lacks effective therapies, unlike HFrEF. Heart failure with mildly reduced ejection fraction (HFmrEF) was excluded due to its overlapping yet distinct pathophysiology and limited representation in current SGLT2 inhibitor trials. With increasing clinical attention on SGLT2 inhibitors and accumulating evidence supporting their use in HFpEF, a comprehensive evaluation of current data is essential. This review focuses exclusively on HFpEF given the limited treatment options available and the growing interest in SGLT2 inhibitors as a potential therapeutic strategy. Although early trials suggest promising results, uncertainties remain regarding their impact on mortality, exercise capacity, and patient-centered outcomes such as symptom relief and quality of life. This systematic review aims to critically evaluate the efficacy and safety of SGLT2 inhibitors in HFpEF, synthesizing available evidence to determine their clinical value. Specifically, it examines their effects on cardiovascular outcomes, functional status, symptom burden, and adverse events in this underserved patient group.

## Review

Method

This review followed the requirements established by Preferred Reporting Items for Systematic Reviews and Meta-Analyses (PRISMA) guidelines [20]. 

Participants, Intervention, Comparison, Outcome (PICO) Framework

The research question was formulated using the PICO framework [21]. Table 1 explains the PICO Framework encompassing the population with HFpEF and SGLT2 inhibitor as intervention while placebo/standard care is a comparative intervention. Studies were included if they enrolled adult patients diagnosed with HFpEF, most commonly defined as left ventricular ejection fraction (LVEF) ≥50%. We accepted trials using established diagnostic criteria, including echocardiographic parameters and clinical symptoms consistent with HFpEF. Studies focusing on HFrEF (LVEF < 40%) or HFmrEF (LVEF 40-49%) were excluded. Additional exclusion criteria included non-randomized designs, non-human studies, or lack of specific SGLT2 inhibitor data.

**Table 1:** PICO framework PICO: Participants, Intervention, Comparison, Outcome; HF: heart failure; HFpEF: heart failure with preserved ejection fraction; SGLT2: sodium-glucose co-transporter 2; SGLT2 inhibitor: sodium-glucose co-transporter 2 inhibitor; MeSH: Medical Subject Headings

Concepts	Text words	Controlled vocabulary
Population/problem patients with heart failure with preserved ejection fraction	“Heart Failure,”, “HFpEF”, “Preserved Ejection Fraction”	"Heart Failure"[Mesh] "HFpEF"[Mesh]
Intervention SGLT2 inhibitor	“SGLT2 Inhibitor, Sodium-Glucose Co-Transporter 2 inhibitors”	"SGLT2 Inhibitor "[MeSH]
Comparative placebo/standard care	“Standard Care, Placebo”	"Standard Care "[MeSH] “Placebo"[Mesh]
Outcomes	“Cardiovascular Death”, “Hospitalization”, “Exercise Capacity”, “quality of life”,	Cardiovascular Death(MeSH), Hospitalization (MeSH), quality of (MeSH)

Research Question

What is the effect of SGLT2 inhibitors on cardiovascular death, hospitalization for heart failure, exercise capacity, symptoms (as measured by the Kansas City Cardiomyopathy Questionnaire (KCCQ)), kidney disease progression, and other renal outcomes in HFpEF?

Search Strategy

A comprehensive literature search was conducted across three databases - PubMed, Embase, and Cochrane Library - to identify relevant studies on SGLT2 inhibitors in HFpEF. The full search strings were adapted for each platform. In PubMed, the search used was ("SGLT2 Inhibitor"[Mesh] OR "Sodium-Glucose Transporter 2 Inhibitors" OR "Empagliflozin" OR "Dapagliflozin" OR "Canagliflozin") AND ("Heart Failure with Preserved Ejection Fraction"[Mesh] OR "HFpEF" OR "Heart Failure, Diastolic" OR "Preserved Ejection Fraction") AND ("Placebo" OR "Standard Care" OR "Control Group"). In Embase, the following terms were used: ('sodium glucose cotransporter 2 inhibitor' OR 'SGLT2 inhibitor' OR empagliflozin OR dapagliflozin OR canagliflozin) AND ('heart failure with preserved ejection fraction' OR HFpEF OR 'diastolic heart failure') AND ('placebo' OR 'standard care' OR 'usual care'). For the Cochrane Library, the search string was ("SGLT2 Inhibitor" OR "Sodium-Glucose Cotransporter 2 Inhibitor" OR empagliflozin OR dapagliflozin OR canagliflozin) AND ("Heart Failure with Preserved Ejection Fraction" OR HFpEF OR "diastolic heart failure") AND ("Placebo" OR "Standard Care"). No restrictions were placed on language or publication date, and reference lists of all included studies were manually screened for additional eligible studies. All search strings were adapted for each database’s indexing and controlled vocabulary (e.g., Emtree for Embase). Additionally, reference lists of included studies and prior systematic reviews were manually screened to identify any potentially missed articles.

Eligibility Criteria

In this review, only experimental studies were included, with varying sample sizes. Studies in which adult patients of either gender having HFpEF were included. The studies focused on hospitalization for heart failure, cardiovascular death, symptoms (as measured by the KCCQ), exercise capacity, kidney disease progression, and other renal outcomes. Studies from the last 10 years, open-access, written in English, and with full-text availability were included in the review. The review excluded all other study designs, such as cohort, case-control, observational studies, case reports, case series, conference abstracts, editorials, letters, review papers, and meta-analyses. Studies on teenagers, children, and animals were also excluded. Studies in which patient outcomes were not related to the role of SGLT2 inhibitors in HFpEF before 2015 were excluded due to restricted data access and incomplete analysis.

Study Selection Process

Initial screening included two independent reviewers reading the articles' titles and abstracts. Then, the two independent reviewers conducted a full-text review by comprehensively reading the articles. Regarding reviewers' disagreement, a consensus was developed [22]. The review included only those studies that were available in full text and met the inclusion criteria.

Methodological Quality Assessment

The methodological quality of the included studies was assessed using the Mixed Methods Appraisal Tool (MMAT) Version 2018. The MMAT assesses five core domains: selection bias, performance bias, detection bias, attrition bias, and reporting bias. A score of 5 indicates high quality (low risk of bias (ROB) in all domains), 4 indicates moderate quality (low risk in most domains but some concerns), and below 4 indicates a potentially high ROB. Two independent reviewers conducted the MMAT assessments. In case of any disagreements, a third reviewer was consulted to achieve consensus. The process ensured objectivity and consistency in the evaluation of study quality. A detailed table (Table 2) has been provided below showing the MMAT score for each study, along with risk ratings across individual bias domains. This allows for a transparent appraisal of methodological rigor and potential limitations [23].

**Table 2:** MMAT scores and bias assessment MMAT: Mixed Methods Appraisal Tool

Study	MMAT score	Selection bias	Performance bias	Detection bias	Attrition bias	Reporting bias	Other bias	Overall risk of bias
Doehner et al., 2024 [26]	4	Low	Low	High	Low	Unclear	None	Moderate
Pitt et al., 2023 [27]	4	Low	Low	High	Low	Unclear	None	Moderate
Anker et al., 2021 [28]	4	Low	Low	High	Low	Unclear	None	Moderate
Solomon et al., 2022 [29]	5	Low	Low	Low	Low	Low	None	Low
Abraham et al., 2021 [30]	5	Low	Low	Low	Low	Low	None	Low
Nassif et al., 2023 [31]	5	Low	Low	Low	Low	Low	None	Low
Spertus et al., 2022 [32]	5	Low	Low	Low	Low	Low	None	Low
Bhatt et al., 2021 [33]	4	Low	Low	High	Low	Unclear	None	Moderate
Herrington et al., 2023 [34]	4	Low	Low	High	Low	Unclear	None	Moderate
Nassif et al., 2019 [35]	5	Low	Low	Low	Low	Low	None	Low
McMurray et al., 2019 [36]	5	Low	Low	Low	Low	Low	None	Low
Packer et al., 2021 [8]	4	Low	Low	High	Low	Unclear	None	Moderate
Sridhar et al., 2024 [37]	5	Low	Low	Low	Low	Low	None	Low
Rådholm et al., 2018 [38]	5	Low	Low	Low	Low	Low	None	Low
Wiviott et al., 2019 [39]	4	Low	Low	High	High	Unclear	None	High
Cosentino et al., 2020 [40]	4	Low	Low	High	Low	Unclear	None	Moderate
Perkovic et al., 2019 [41]	5	Low	Low	Low	Low	Low	None	Low

Data Extraction and Synthesis

Data extraction for this systematic review was conducted using a standardized, pilot-tested form developed in Microsoft Excel 365 (Microsoft Corporation, Redmond, WA, USA). Two independent reviewers extracted data in parallel after completing calibration exercises on three randomly selected studies to ensure consistency in interpretation and minimize variability. Disagreements were resolved through discussion, with arbitration by a third reviewer when necessary. The data extraction form was pilot-tested on five included studies and refined for clarity and completeness. Extracted data were organized using Covidence (Covidence Pty Ltd, Melbourne, Australia) for screening and reviewer assignment. The extracted variables were clearly defined and included the following: study objectives (focused on the effects of SGLT2 inhibitors in heart failure with preserved ejection fraction [HFpEF]), study design and population characteristics (e.g., randomized controlled trials (RCTs), sample size, ejection fraction thresholds, New York Heart Association (NYHA) class), intervention details (e.g., type and dose of SGLT2 inhibitor), comparators (placebo or standard of care), outcomes measured (e.g., cardiovascular mortality, heart failure hospitalization, KCCQ scores, six-minute walk test), statistical methods used for survival analysis (e.g., hazard ratios, confidence intervals), key findings (clinically and statistically significant effects of SGLT2 inhibitors), prognostic indicators (e.g., age, N-terminal pro-B-type natriuretic peptide (NT-proBNP), baseline EF), and reported complications or adverse effects associated with therapy. This comprehensive and transparent process ensured rigorous and reproducible data synthesis aligned with PRISMA guidelines [24].

In this review of SGLT2 inhibitors in HFpEF, we applied a structured thematic synthesis to organize and interpret outcome data across trials with diverse designs, populations, and endpoints. This approach was chosen to synthesize evidence from RCTs that reported similar types of outcomes (e.g., hospitalization rates, KCCQ scores, and NT-proBNP levels) but used varying measures, follow-up durations, and patient subgroups. The process followed four clearly defined steps: (1) Initial coding-two reviewers independently reviewed and coded quantitative results (e.g., hazard ratios, mean differences) into outcome categories (e.g., cardiovascular death, quality of life, exercise capacity); (2) Theme development-codes were grouped into higher-order themes aligned with clinical domains such as symptom burden, functional status, and healthcare utilization; (3) Theme refinement and validation-themes were reviewed jointly by the reviewers, inconsistencies were resolved through discussion, and themes were refined to avoid overlap or redundancy; and (4) Final synthesis-themes were structured narratively and supplemented with comparative tables to allow cross-study interpretation. This thematic synthesis approach enabled a transparent and clinically relevant integration of findings across trials without overreliance on statistical pooling.

Ethical Consideration

As this study is a systematic review of previously published literature and involves no direct human or animal subjects, formal ethical approval was not required. No personal or identifiable data were collected, and issues of confidentiality or anonymity do not apply. The review was conducted with transparency and rigor, adhering to PRISMA guidelines to ensure reproducibility.

Results

**Figure 1:**
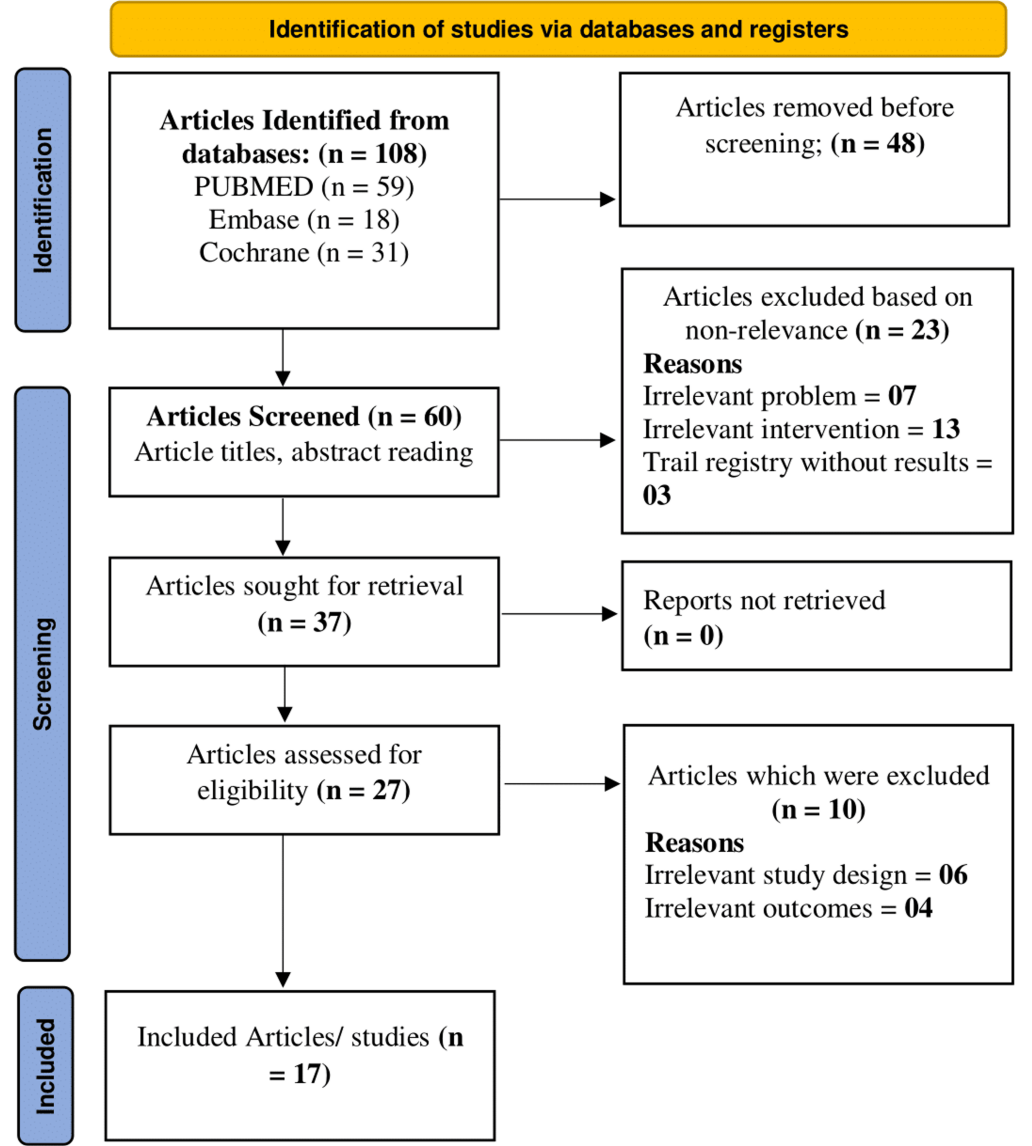
PRISMA flow chart PRISMA: Preferred Reporting Items for Systematic Reviews and Meta-Analyses

The study selection process adhered to the PRISMA 2020 guidelines and is detailed in Figure 1. A total of 108 articles were initially identified through database searches (PubMed = 59, Embase = 18, Cochrane = 31). After removing 48 duplicates, 60 articles were screened based on titles and abstracts. Twenty-three studies were excluded due to irrelevance either to the problem (n = 7), intervention (n = 13), or due to unlinked trial registries (n = 3). Thirty-seven articles were retrieved, and 27 full texts were assessed for eligibility. Ten studies were excluded due to irrelevant design (n = 6) or outcomes (n = 4). Finally, 17 studies were included in the review after quality assessment, with scores distributed as high (n = 10), moderate (n = 6), and low (n = 1). Although the review was not registered in PROSPERO, a protocol was prepared beforehand to guide the methodology.

*MMAT ROB* *Ratings by Study and Bias Domain*

Table 2 explains the ROB calculated by the MMAT [25]. MMAT Score 4 indicates a medium ROB, and MMAT Score 5 indicates a low ROB. The low risk showed no significant issues related to bias in the study. High risk shows potential biases that may impact the study's internal validity. An unclear score shows the lack of sufficient information to assess the ROB in that domain. Ten studies had low ROB and were classified as "high quality." Seven had uncertain ROB, lowering the evidence by one point to "moderate quality," while one study had a high ROB.

The methodological quality of all included studies was assessed using the MMAT, 2018 version. Two independent reviewers evaluated each study across six domains: selection bias, performance bias, detection bias, attrition bias, reporting bias, and other sources of bias. Discrepancies were resolved through discussion and consensus. Out of 17 studies assessed, nine were rated as high quality (MMAT score = 5), and eight studies received a score of 4, indicating a moderate ROB in one domain. Notably, selection bias and performance bias were consistently low across all studies, reflecting adequate randomization and appropriate delivery of interventions. However, detection bias was the most frequent source of concern, marked as high in eight studies largely due to the lack of clear blinding procedures for outcome assessors. Reporting bias was classified as “unclear” in seven studies, where study protocols or prespecified outcomes were not sufficiently documented to rule out selective reporting. Attrition bias was mostly low, with only one study rated high due to considerable loss to follow-up. None of the studies showed concerns under "other bias." The overall ROB was considered low in nine studies, moderate in seven studies, and high in one study [8,26-36,37-41].

Characteristics and Findings of Studies Included in the Review

Table 3 describes a series of RCTs, including double-blind and multinational designs, that have investigated the efficacy of various SGLT2 inhibitors in patients with HFpEF, often including those with or without T2DM and chronic kidney disease (CKD). Doehner et al. worked on one double-blind RCT that enrolled 5,988 adults with chronic heart failure (NYHA class II-IV) and LVEF > 40%, with elevated NT-proBNP levels, comparing empagliflozin 10 mg once daily to placebo over a median follow-up of 26.2 months [26]. Similarly, multinational RCTs with large sample sizes (n = 5,988 and n = 6,263) evaluated empagliflozin and dapagliflozin, respectively, in HFpEF patients, both showing beneficial outcomes with daily oral 10 mg doses [28,29]. Other smaller-scale RCTs (n = 312 and n = 324) compared empagliflozin and dapagliflozin in HFpEF patients with LVEF ≥ 50% and ≥ 45%, respectively [30,31]. A decentralized RCT (n = 448) examined canagliflozin 100 mg in heart failure patients with LVEF ≥ 45% [32], while another RCT (n = 1,222) tested sotagliflozin in T2DM patients hospitalized for worsening heart failure with LVEF ≥ 50% [33]. Additional trials included empagliflozin in CKD patients (n = 6,609), dapagliflozin in heart failure patients (n = 263 and n = 4,744), and empagliflozin in 3,730 HFpEF patients [8,34-36]. Large-scale RCTs evaluated sotagliflozin (n = 10,584), canagliflozin (n = 10,142), dapagliflozin (n = 17,160), ertugliflozin (n = 8,246), and canagliflozin (n = 4,401) in T2DM patients with or without CKD and HFpEF, reinforcing the potential role of SGLT2 inhibitors across a range of comorbid conditions [37-41].

**Table 3:** Summary of included studies HFpEF: heart failure with preserved ejection fraction; HFrEF: heart failure with reduced ejection fraction; HF: heart failure; LVEF: left ventricular ejection fraction; NYHA: New York Heart Association; NT-proBNP: N-terminal pro-B-type natriuretic peptide; T2DM: type 2 diabetes mellitus; CKD: chronic kidney disease; CV: cardiovascular; HR: hazard ratio; CI: confidence interval

Author/year	Objectives	Study design/sample size	Study population	Study groups	Intervention protocol	Tools/outcomes	Study findings
Doehner et al., 2024 [26]	To investigate the effect of empagliflozin on serum uric acid (SUA in patients with HFpEF)	Double-blind randomized controlled trial; n = 5,988	Adults with chronic HF (NYHA class II-IV) and LVEF > 40%, with elevated NT-proBNP levels	Treatment with empagliflozin vs. placebo	Empagliflozin 10 mg orally once daily group (n ≈ 2,962); placebo group (n ≈ 2,962) median follow-up: 26.2 months	Primary: Composite of CV death or hospitalization for HF (HHF)	Empagliflozin reduced SUA significantly (by −0.99 mg/dL at 4 weeks); reduced hyperuricemic events by 38% (HR: 0.62, p < 0.0001)
Pitt et al., 2023 [27]	To evaluate the efficacy of sotagliflozin versus placebo in reducing mortality and HF-related events in HFpEF	RCT; total n = 290 sotagliflozin and 306 placebo	-	Sotagliflozin group - placebo group	once-daily sotagliflozin 200 mg (with a possible dose escalation to 400 mg) or placebo	Main outcome: CV death or HF-related events (hospitalization or urgent care) within 30 and 90 days post-discharge	Significant reduction in the primary outcome at both 30 days (HR: 0.49, p = 0.023) and 90 days (HR: 0.54, p = 0.004) with sotagliflozin. Reduced all-cause mortality at 90 days (HR: 0.39, p = 0.024)
Anker et al., 2021 [28]	Evaluate empagliflozin's effect on CV death or HF hospitalization in HFpEF	Multinational RCT; n = 5,988	HFpEF patients (LVEF > 40%) with/without diabetes	Empagliflozin 10 mg vs. placebo	Daily oral empagliflozin 10 mg	Primary: CV death or HF hospitalization	Empagliflozin significantly reduced the risk of the primary composite outcome compared to placebo (HR = 0.79; 95% CI: 0.69-0.90; p < 0.001). The benefit was primarily driven by a 29% reduction in HF hospitalization
Solomon et al., 2022 [29]	Assess dapagliflozin's efficacy in HFpEF	Multinational RCT; n = 6,263	HFpEF patients (LVEF > 40%) with/without diabetes	Dapagliflozin 10 mg vs. placebo	Daily oral dapagliflozin 10 mg	Primary: CV death or HF hospitalization	Dapagliflozin significantly reduced the risk of the primary composite outcome compared to placebo (HR = 0.82; 95% CI: 0.73-0.92; p < 0.001). Benefits were consistent across the LVEF spectrum
Abraham et al., 2021 [30]	Evaluate empagliflozin's impact on exercise capacity in HFpEF	RCT; n = 312	HFpEF patients (LVEF ≥ 50%)	Empagliflozin 10 mg vs. placebo	Daily oral empagliflozin 10 mg	Six-minute walk test distance	No significant difference in exercise capacity between groups (mean difference: 0.0 meters; 95% CI: -14.2 to 14.2; p = 0.99)
Nassif et al., 2023 [31]	Assess dapagliflozin's effect on symptoms and physical limitations in HFpEF	RCT; n = 324	HFpEF patients (LVEF ≥ 45%)	Dapagliflozin 10 mg vs. placebo	Daily oral dapagliflozin 10 mg	Kansas City Cardiomyopathy Questionnaire (KCCQ) scores	Dapagliflozin significantly improved KCCQ clinical summary score by 5.8 points compared to placebo (95% CI: 2.3-9.2; p = 0.001)
Spertus et al., 2022 [32]	Evaluate canagliflozin's effect on HF symptoms in HFpEF	Decentralized RCT; n = 448	HF patients (LVEF ≥ 45%)	Canagliflozin 100 mg vs. placebo	Daily oral canagliflozin 100 mg	KCCQ total symptom score	Canagliflozin significantly improved KCCQ total symptom score by 4.3 points compared to placebo (95% CI: 0.8-7.8; p = 0.016).
Bhatt et al., 2021 [33]	Assess sotagliflozin's efficacy in worsening HF including HFpEF	RCT; n = 1,222	T2DM patients hospitalized for worsening HF (LVEF ≥ 50%)	Sotagliflozin vs. placebo	Daily oral sotagliflozin	CV death, HF hospitalization, urgent visits	Sotagliflozin significantly reduced the composite endpoint of CV death, HF hospitalization, and urgent visits (HR = 0.67; 95% CI: 0.52-0.85; p < 0.001)
Herrington et al., 2023 [34]	Evaluate empagliflozin's effect on renal outcomes; included HFpEF patients	RCT; n = 6,609	CKD patients, some with HFpEF	Empagliflozin 10 mg vs. placebo	Daily oral empagliflozin 10 mg	Kidney disease progression, CV death	Empagliflozin significantly reduced the risk of kidney disease progression or CV death (HR = 0.72; 95% CI: 0.64-0.82; p < 0.001)
Nassif et al., 2019 [35]	Assess dapagliflozin's effect on natriuretic peptides and symptoms in HFrEF and HFpEF	RCT; n = 263	HF patients (LVEF ≥ 40%)	Dapagliflozin 10 mg vs. placebo	Daily oral dapagliflozin 10 mg	NT-proBNP levels, KCCQ scores	No significant change in NT-proBNP levels; however, dapagliflozin improved KCCQ scores by 5.3 points compared to placebo (95% CI: 1.3-9.3; p = 0.01)
McMurray et al., 2019 [36]	Evaluate dapagliflozin's effect on worsening HF or CV death in HFrEF and HFpEF	RCT; n = 4,744	HF patients (LVEF ≥ 40%)	Dapagliflozin 10 mg vs. placebo	Daily oral dapagliflozin 10 mg	CV death, HF hospitalization	Dapagliflozin significantly reduced the risk of the primary composite outcome (HR = 0.74; 95% CI: 0.65-0.85; p < 0.001).
Packer et al., 2020 [8]	Assess empagliflozin's efficacy in HFrEF and HFpEF	RCT; n = 3,730	HF patients (LVEF ≥ 40%)	Empagliflozin 10 mg vs. placebo	Daily oral empagliflozin 10 mg	CV death, HF hospitalization	Empagliflozin significantly reduced the risk of the primary composite outcome (HR = 0.75; 95% CI: 0.65-0.86; p < 0.001)
Sridhar et al., 2024 [37]	Evaluate sotagliflozin's effect on CV outcomes in T2DM with CKD; included HFpEF patients	RCT; n = 10,584	T2DM patients with CKD, some with HFpEF	Sotagliflozin vs. placebo	Daily oral sotagliflozin	CV death, HF hospitalization	Sotagliflozin significantly reduced the risk of the composite endpoint of CV death, HF hospitalization, and urgent visits (HR = 0.74; 95% CI: 0.63-0.88; p < 0.001)
Rådholm et al., 2018 [38]	Assess canagliflozin's CV safety; included HFpEF patients	RCT; n = 10,142	T2DM patients, some with HFpEF	Canagliflozin vs. placebo	Daily oral canagliflozin	CV events, HF hospitalization	Canagliflozin reduced the risk of HF hospitalization (HR = 0.67; 95% CI: 0.52-0.87; p = 0.002)
Wiviott et al., 2019 [39]	Evaluate dapagliflozin's CV outcomes; included HFpEF patients	RCT; n = 17,160	T2DM patients, some with HFpEF	Dapagliflozin 10 mg vs. placebo	Daily oral dapagliflozin 10 mg	CV death, HF hospitalization	Dapagliflozin significantly reduced the risk of HF hospitalization (HR = 0.73; 95% CI: 0.61-0.88; p = 0.0004)
Cosentino et al., 2020 [40]	Assess ertugliflozin's CV safety; included HFpEF patients	RCT; n = 8,246	T2DM patients, some with HFpEF	Ertugliflozin vs. placebo	Daily oral ertugliflozin	CV events, HF hospitalization	Ertugliflozin significantly reduced the risk of HF hospitalization (HR = 0.70; 95% CI: 0.54-0.90; p = 0.006)
Perkovic et al., 2019 [41]	Evaluate canagliflozin's renal and CV outcomes; included HFpEF patients	RCT; n = 4,401	T2DM patients with CKD, some with HFpEF	Canagliflozin vs. placebo	Daily oral canagliflozin	Renal outcomes, CV death, HF hospitalization	Canagliflozin significantly reduced the risk of HF hospitalization (HR = 0.61; 95% CI: 0.47-0.80; p < 0.001)

Cardiovascular death: The main goal of heart failure management is to prevent cardiovascular death, as it represents the most serious consequence of heart disease. Doehner et al.'s study indicates that SGLT2 inhibitors deliver substantial benefits against cardiovascular death among patients with heart failure [26]. Solomon et al. demonstrated that dapagliflozin effectively reduced cardiovascular mortality among HFrEF and HFpEF patients [29]. Anker et al. revealed that empagliflozin had a positive impact on reducing cardiovascular mortality in HFrEF patient groups [28]. Pitt et al. show that particular therapeutic approaches successfully reduce mortality risks in heart failure patients and enhance their survival durations [27].

Hospitalization for heart failure: The occurrence of hospital admissions for heart failure acts as a key indicator to monitor progressive heart disease and worsening symptoms in heart failure patients. Solomon et al. indicated that repeated need for hospitalization due to heart failure results in increased healthcare expenses and stronger medical challenges for patient populations [29]. Abraham et al. confirm that particular treatments produce noteworthy reductions in hospital admission rates [30]. The research by McMurray et al. established empagliflozin's ability to shield HFpEF patients from hospitalizations, yet Solomon et al.'s study established dapagliflozin's effectiveness specifically in HFrEF patients [29,36]. Medical research reveals that SGLT2 inhibitors function as essential healthcare tools that stop hospitalizations due to worsening heart failure conditions. The study by Pitt et al. in 2023 demonstrated how sotagliflozin led patients to prevent urgent heart failure visits and decrease hospital stay duration, thus proving emerging treatments benefit patient health [27].

Exercise capacity: The assessment of exercise capacity by healthcare professionals using the six-minute walk test measurements enables them to determine both patient mobility and patient quality of life. Different research studies present contradictory findings about how therapies affect exercise capacity measurements. The study by McMurray et al. in 2019 showed that empagliflozin treatment had no meaningful impact on six-minute walk distance exercise capacity [36]. According to Solomon et al., dapagliflozin failed to produce quantifiable changes in exercise capabilities [29]. Herrington et al. show that heart failure symptom reduction alongside hospital admission prevention from SGLT2 inhibitors does not translate into enhanced exercise capacity for patients [34]. The hospitalization rates show improvement, but this data does not always translate to enhanced exercise capacity among heart failure patients, despite exercise capacity playing an important role in heart failure treatment.

KCCQ total symptom score: The assessment of exercise capacity by healthcare professionals using the six-minute walk test measurements enables them to determine both patient mobility and patient quality of life. Different research studies present contradictory findings about how therapies affect exercise capacity measurements. Empagliflozin treatment produced no substantial changes in six-minute walk distance exercise capacity, according to McMurray et al. and Solomon et al., which established that dapagliflozin treatment had no meaningful effect on exercise function [29,36]. The beneficial effects of SGLT2 inhibitors demonstrated in heart failure symptom reduction and hospital admission prevention do not necessarily yield improvements in patient exercise capacity. Rates of hospitalization demonstrate improvement, but these results do not necessarily lead to better exercise capacity for heart failure patients, even though exercise capacity remains vital for heart failure therapy.

Kidney disease progression: Nassif et al. and Spertus et al. showed that dapagliflozin and empagliflozin demonstrated their capacity to prevent kidney disease progression among heart failure patients. Heart failure hospitalization rates decreased, together with protection against kidney function decline, as the medications blocked the decrease in estimated glomerular filtration rate (eGFR) [32,35]. Perkovic et al.'s research findings demonstrated that canagliflozin exhibited a superior ability to minimize kidney disease progression, which underscores the necessity of kidney protection during heart failure medical care [41]. Wiviott et al. showed that SGLT2 inhibitors provide essential benefits for treating both cardiovascular problems and kidney diseases at the same time [39].

Renal outcomes: Heart failure treatments for patients with kidney disease require complete renal outcome assessments to monitor disease development alongside kidney protection evaluation. The research by Cosentino et al. evaluated dapagliflozin as a therapeutic option to improve kidney outcomes in patients who have CKD alongside heart failure. The research indicated that dapagliflozin reduced patients’ chances of developing kidney failure and cardiovascular death, experiencing heart failure-related hospital stays, and sustaining kidney damage [40]. Sridhar et al. found that canagliflozin can help prevent diabetes patients with CKD from worsening their kidney disease [37]. The study from Herrington et al. revealed that using SGLT2 inhibitors can save the kidneys and benefit the cardiovascular system, so heart failure patients could receive this potential treatment [34].

Discussion

Cardiovascular death is a critical endpoint in heart failure trials, particularly for patients with HFpEF, as it reflects disease severity and long-term prognosis. Rådholm et al. evaluated the effects of canagliflozin in patients with type 2 diabetes and heart failure, including HFpEF, and reported a significant reduction in cardiovascular death. This supports the potential of SGLT2 inhibitors to improve survival in this subgroup. By contrast, Aguiar-Neves et al. found that liraglutide, a glucagon-like peptide 1 (GLP-1) receptor agonist, did not demonstrate similar cardiovascular benefits in HFpEF patients. Furthermore, Brust-Sisti et al. showed that the cardioprotective effects of empagliflozin and dapagliflozin surpassed those of other antidiabetic agents, regardless of ejection fraction status. These benefits appear consistent across both diabetic and non-diabetic HFpEF patients, as reported in recent subgroup analyses [38,42,43].

Hospitalizations for heart failure are commonly used endpoints reflecting morbidity, healthcare utilization, and disease trajectory. Several trials, including those by Cosentino et al., Girerd and Zannad, and Mentz et al., consistently demonstrated that SGLT2 inhibitors reduce heart failure hospitalizations in both HFpEF and HFrEF cohorts. These benefits are partly mediated through improvements in blood pressure control, volume status, and renal function. Mechanistically, SGLT2 inhibitors modulate natriuresis, reduce interstitial fluid overload, and attenuate sympathetic activation. Preclinical data further suggest they exert anti-inflammatory and anti-fibrotic effects by reducing NLRP3 inflammasome activity and transforming growth factor-beta (TGF-β) signaling, mechanisms that contribute to diastolic dysfunction in HFpEF [40,44,45].

Functional capacity, assessed via the six-minute walk test, has yielded inconsistent results in HFpEF studies. For instance, Spertus et al. found no significant improvement in six-minute walk test distance with sacubitril/valsartan, despite symptomatic relief. Similarly, empagliflozin showed modest or no improvements in exercise capacity in trials such as those by Chen et al., while Deschaine B et al. and Keller et al. noted that although cardiovascular outcomes improved, gains in exercise capacity were limited. These findings reflect the complex, multifactorial nature of HFpEF, where comorbidities such as obesity, sarcopenia, insulin resistance, and microvascular dysfunction may restrict functional gains despite optimized hemodynamics [32,46-48].

Quality of life and symptom burden are effectively captured by the KCCQ. Data from Packer showed that empagliflozin, dapagliflozin, and sotagliflozin each led to significant improvements in KCCQ scores. These findings are consistent with those of McMurray et al., who observed similar benefits with sacubitril/valsartan, reinforcing that multiple pharmacologic agents can meaningfully improve patient-reported outcomes in HFpEF. Notably, these benefits were seen in both diabetic and non-diabetic patients, although some studies suggested greater effect sizes in those with comorbid type 2 diabetes, likely due to enhanced natriuretic and metabolic responses [8,36,49,50].

Renal function preservation is a central concern in managing HFpEF, especially given the high overlap with CKD. Trials such as those by Nassif et al. demonstrated that dapagliflozin and canagliflozin slow eGFR decline and reduce renal event incidence. Palazzuoli et al. and Salvatore et al. provided further evidence that SGLT2 inhibitors confer kidney protection comparable to or exceeding that of angiotensin-converting enzyme (ACE) inhibitors or angiotensin receptor blockers (ARBs). These effects are believed to arise from reduced intraglomerular pressure, improved tubular-glomerular feedback, and anti-fibrotic renal signaling, alongside blood pressure and glycemic control [35,51-53].

For patients with HFpEF and advanced CKD, renal outcomes such as progression to end-stage kidney disease (ESKD) or dialysis are critical. Bhatt et al. demonstrated that canagliflozin substantially reduced CKD progression and dialysis risk in diabetic patients. Similar renal benefits were observed in Siddiqi et al., who reported protective effects of both canagliflozin and dapagliflozin in HFpEF patients with CKD. Conversely, Savage et al. found that finerenone, though beneficial, did not offer the same level of integrated cardiovascular-renal protection as SGLT2 inhibitors, reinforcing their preferential role in such comorbid populations [33,54-56].

Importantly, subgroup analyses have highlighted nuanced treatment responses. SGLT2 inhibitors appear to be more effective in patients with diabetes or with LVEF in the lower HFpEF range (e.g., 41-49%), where cardiac remodeling and congestion predominate. In patients with higher LVEF (>60%), the benefits are less pronounced, suggesting a possible dose-response relationship with LVEF or differing pathophysiologic drivers in stiff ventricles versus volume-overloaded hearts. Further stratified analyses are needed to refine patient selection and optimize therapeutic gain.

Several limitations were identified across the included studies, particularly in the definition and diagnosis of HFpEF. Variability in ejection fraction thresholds, diagnostic criteria, and inclusion parameters created heterogeneity, complicating direct comparisons and synthesis. Additionally, patient populations differed significantly in terms of age, comorbidities (such as diabetes and CKD), and baseline functional status, potentially influencing treatment outcomes. These inconsistencies underscore the need for standardized HFpEF definitions and more uniform eligibility criteria in future trials to enhance comparability and clinical applicability. There is a clear need for further high-quality RCTs to validate the current findings. Future research should involve large-scale, long-term RCTs that assess treatment outcomes across diverse demographic groups, particularly those with multiple comorbidities. Additionally, studies should explore the synergistic effects of combining pharmacological interventions with non-pharmacologic strategies, such as structured physical rehabilitation programs. Expanding the therapeutic evidence base in this manner will support more personalized treatment approaches and ultimately improve clinical outcomes for patients with HFpEF.

## Conclusions

SGLT2 inhibitors have emerged as essential therapeutic agents in the management of HFpEF, offering multifaceted benefits. These include reductions in cardiovascular mortality, decreased hospital admission rates, improved patient-reported symptoms, and protection of renal function. However, despite these advantages, their effects on exercise capacity remain limited, highlighting the need for adjunctive therapies aimed at enhancing physical function. Ongoing research is crucial to fully understand the long-term benefits and optimal use of SGLT2 inhibitors in HFpEF treatment. Emphasizing the need for further high-quality RCTs, future investigations should focus on diverse patient populations, long-term outcomes, and the integration of pharmacologic and non-pharmacologic strategies to optimize care and improve overall patient prognosis.
